# Changes in diet quality during a 12 month weight loss randomised controlled trial

**DOI:** 10.1186/s40795-017-0157-z

**Published:** 2017-04-17

**Authors:** Cinthya Wibisono, Yasmine Probst, Elizabeth Neale, Linda Tapsell

**Affiliations:** 1 School of Medicine, Faculty of Science, Medicine and Health, University of Wollongong, Wollongong, New South Wales 2522 Australia; 2 Illawarra Health and Medical Research Institute, University of Wollongong, Wollongong, New South Wales 2522 Australia

**Keywords:** Diet quality, Dietary patterns, Weight loss, Trial, Index, Walnuts

## Abstract

**Background:**

Reductions in energy intake are seen in weight loss trials, but whether this occurs with improvements to diet quality (DQ) is less established. The aim of this study was to evaluate changes in diet quality in a sample of volunteers in a weight loss trial.

**Methods:**

This was a secondary analysis of dietary data from a lifestyle intervention trial (the HealthTrack study) which advised on dietary guidelines. The trial ran for 12 months with three treatment groups: control (general advice *C*), intervention (individualised advice, *I*), and intervention plus a supplement of walnuts (*IW*). Both the published *a priori diet quality score* (APDQS, maximum score 164) and a study specific Diet Quality Tracker (DQT, maximum score 44) indicated compliance to dietary advice. DQ scores calculated at 0, 3months and 12months were evaluated using two-way RMANOVA, one-way ANOVA and one-way RMANOVA. Changes in intakes of food groups and nutrients were analysed using Kruskal-Wallis and Friedman’s tests.

**Results:**

There were no differences between groups at baseline, but at 3months *IW* recorded higher DQ scores (APDQS:96 ± 10; DQT:22 ± 5, *P* < 1 × 10^−3^ for both) compared to *I* (APDQS:91 ± 13, *P* < 1 × 10^−3^; DQT:21 ± 4, *P* < 1 × 10^−2^) and *C* (APDQS:87 ± 12, *P* < 5 × 10^−2^; DQT:19 ± 4, *P* > 5 × 10^−2^), and a higher consumption of nuts at 3 months (*P* < 1 × 10^−3^), and 12months (*P* < 1 × 10^−2^). All groups reported decreased intakes of discretionary foods/beverages assessed by the DQT (*P* < 1 × 10^−3^ for *IW* and *I*; *P* < 1 × 10^−2^ for *C*). The APDQS showed this as reduced intakes of grain based desserts (*P* < 1 × 10^−3^ at 3 and 12months), and salty snacks at 12months (*P* < 1 × 10^−3^ for *IW* and *I*; *P* < 5 × 10^−2^ for *C*). Intakes of monounsaturated and saturated fatty acids were lowest, and polyunsaturated fatty acids highest for *IW* (*P* < 1 × 10^−3^), resulting in a higher dietary polyunsaturated:saturated fat ratio (*P* < 1 × 10^−3^).

**Conclusions:**

Lifestyle intervention addressing dietary guidelines can lead to significant reductions in consumption of discretionary foods and saturated fat, but individualised advice may have a greater impact on improving overall DQ regardless of DQI used. Providing a healthy food supplement may help assure higher DQ and where the food is walnuts, produce commensurate differences in dietary fatty acid profiles.

**Trial registration:**

ANZCTRN 12614000581662. Date of registration: 30th May 2014.

**Electronic supplementary material:**

The online version of this article (doi:10.1186/s40795-017-0157-z) contains supplementary material, which is available to authorized users.

## Background

Obesity is a complex issue with multiple influential factors contributing to its cause [1, 2], but energy balance lies at the core [3, 4]. When energy intake, derived from consumption of foods and beverages, exceeds energy expended, changes in body weight inevitably occur leading to a state of overweight or obesity [1]. Reducing energy intake should result in weight loss. When energy intake is reduced, however, the quality of foods included in the diet is important. Lowering overall energy intake must not compromise the nutritional adequacy of diets [5–7]. To avoid this from occurring, concerted efforts should be placed on choosing nutrient dense foods [7]. Replacing “non-core”, “discretionary” or energy-dense foods with little nutritional value [8] with nutrient-dense “core” foods, helps to ensure diet quality is maintained when dietary prescriptions are developed for weight loss [9].

Diet quality (DQ) is a central parameter of dietary pattern research. The DQ concept is used to evaluate the diets of populations or individuals [10–12], assess the effectiveness of nutrition interventions [13] and expose diet-disease associations [14]. Arising largely from epidemiological studies, the DQ concept encapsulates descriptors related to nutrition, such as, “healthy diet, balanced diet, nutritious food, etc.” [15]. A DQ score is dependent on individual food choices. As dietary change involves a substitution and compensatory effect influencing more than one food [16], it is foreseeable that DQ may also change. The concept of food synergy, which acknowledges the interface between foods, nutrients and dietary patterns, provides a framework for considering these changes in analyses related to health outcomes [17, 18]. Recognised complexities emerging from nutrition epidemiology [19–21] have exposed the necessity for a more integrated approach utilising diet quality indices (DQIs) to examine the ‘whole of diet’ effects [22]. Several reviews [12, 14, 23–27] have identified multiple DQIs that vary in design and the referent food or nutrient characteristics. DQIs tend to be applied in epidemiological studies to establish diet-disease links, characterise patterns of food consumption or evaluate adherence to country-specific dietary guidelines [28–31]. Characteristically, they are developed with reference to dietary guidelines, utilising an *a priori* approach [16]. For example, in Australia, the Australian Dietary Guidelines (ADG) and the Australian Guide to Healthy Eating (AGHE) [8] both emphasise a set of nutrient-rich ‘core’, or non-discretionary foods, on which recommended daily meals and snacks are based, and an alternate list of nutrient-poor ‘discretionary’ foods and beverages intended for occasional consumption. Both the ADG and the AGHE have been utilised by DQIs applied in Australian population samples [32–35].

In clinical settings, DQIs can address questions relating to changing dietary patterns on trial outcomes, and in these cases simple dietary assessment tools are preferred [36]. The data is evaluated in terms of adherence to a particular dietary pattern, such as a Mediterranean diet [37, 38] or to assess dietary behaviour change resulting from an intervention [39]. The tools can resemble questionnaires which enable a score to be derived, to distinguish between individuals with poor versus high standards of DQ [40]. Three randomised controlled trials (RCT) [38, 41, 42] were identified, applying purpose specific DQIs to assess dietary patterns likely to emerge in the study setting, all of which were context sensitive. DQIs developed independent of the context of a study may also be applied in other study settings, but there may be issues with food categorisation. For example, the *a priori diet quality score* (APDQS), developed within population-based studies [43–46], has been used to evaluate changes in dietary patterns in an intervention trial [47]. In the current study, the food categories applied to generate this score would not cover all foods included in dietary guidelines advice specific to this trial, but it would cover some of the food categories. The aim of the study reported here was to evaluate changes in diet quality by means of two DQIs, amongst participants in the HealthTrack study, a lifestyle intervention trial targeting weight loss in the Australian context. For this analysis, the APDQS, a validated DQI developed to define a healthy dietary pattern protective of cardiovascular disease (CVD) and a study specific Diet Quality Tracker (DQT) were applied.

## Methods

### Study context: The HealthTrack study

A secondary analysis of data from the HealthTrack study, a 12 month RCT was performed [48]. In brief, the primary hypothesis of the HealthTrack study was that a novel interdisciplinary approach to individualised lifestyle intervention (*I*) will result in greater weight loss compared to usual care (*C*). The primary outcome of the HealthTrack study was body weight; secondary outcomes included disease risk factors such as serum lipids, glucose and blood pressure, as well as behaviour relating to diet, activity and psychological factors. Participants randomised to a second intervention arm were provided with a healthy food supplement, 30 g of walnuts, intended for daily consumption for the duration of the study (*IW*). All groups received the same intensity of intervention, attending the clinic for individual consultations for an intensive first phase (0, 1, 2, 3 months) followed by a less intensive follow up phase (6, 9, 12 months), but the *I* and *IW* groups were given more specific dietary advice by a dietitian. A total of *n* = 377 participants were recruited for the study through advertisements across a variety of avenues including local newspapers, health services channels, primary healthcare networks and workplace environments. From the *n* = 377 who were recruited and randomised, *n* = 157 completed the study. The analysis reported here was restricted to completers only. Ethics approval was provided by the University of Wollongong/Illawarra Shoalhaven Local Health District Human Research Ethics Committee (Health and Medical) (HE 13/189) and the study was registered with the Australian and New Zealand Clinical Trial Registry (ANZCTRN 12614000581662). Informed written consent was obtained from all participants prior to commencing the study.

### Dietary data

Dietary intake from the HealthTrack study was assessed via diet history (DH) [49] interviews facilitated by Accredited Practising Dietitians (APDs). Dietary assessments were conducted during clinic visits at baseline, and subsequently every quarter over a 12 month period. Dietary data was entered into FoodWorks dietary analysis software (Version 7, 2012, Xyris Software, Spring Hill, QLD, Australia) using the Australian Food and Nutrient (AUSNUT) 2007 food composition database [50], the most up-to-date food composition database at the time of the study commencement. To utilise the most recent food group classifications, including ‘discretionary’ food and beverage categories, the dietary data required conversion to the more recent AUSNUT 2011-13 food composition database [51] to apply the nested hierarchical food and beverage group classification system [52], originally developed to analyse dietary data from the 2011-12 National Nutrition and Physical Activity Survey (NNPAS) [53], a component of the Australian Health Survey (AHS). Dietary data collected during the HealthTrack study was converted to AUSNUT 2011-13 using a systematic method described in detail, elsewhere [54].

### The Diet Quality Tracker (DQT)

The DQT (Table 1) was developed for the context of the HealthTrack study. Diet information sheets developed for the HealthTrack study provided the basis for categorising foods for the purposes of scoring. The food groups in the DQT (Table 2) also represented the major ‘core’, or nutrient rich, food groups in the AGHE [8] making them suitable for assessing diet quality. An extension involved separating out the AGHE vegetable food group into three groups: vegetables, ‘starchy’ vegetables and legumes to better reflect the dietary advice provided in the HealthTrack study where starchy vegetables were limited to one serve per day, and legumes were emphasised for their associations with successful weight loss [55, 56]. Discretionary foods and beverages were initially separated into ‘alcoholic beverages’ and ‘discretionary foods/beverages’ as identified using the NNPAS Discretionary Food List [57]. Given the study design, consumption of all nuts/seeds reported was excluded from the DQT. The final DQT consisted of ten food groups, eight representing ‘core’ food groups and two representing ‘discretionary’ items.

**Table 1:** Food groups and daily consumption standards in the Diet Quality Tracker

Food group	Point awarded	Serve size equivalent	Single serve equivalent (kJ)	Justification^a^
Non starchy vegetables	0	<1	80	Upper limit represents the minimum number of serves recommended in the ADG.
	1	≥1 < 2		
	2	≥2 < 3		
	3	≥3 < 4		
	4	≥4 < 5		
	5	≥5		
Grains	0	<1	335	Upper limit represents the minimum number of serves recommended in the ADG.
	1	≥1 < 2		
	2	≥2 < 3		
	3	≥3 < 4		
	4	≥4 < 5		
	5	≥5		
Fruit	0	0	285	Upper limit represents the minimum number of serves recommended in the ADG.
	1	>0 < 0.5		
	2	≥0.5 < 1		
	3	≥1 < 1.5		
	4	≥1.5 < 2		
	5	≥2		
Legumes	0	0	335	Evidence of association for weight loss [55, 56]
	1	>0 < 0.25		
	2	≥0.25 < 0.5		
	3	≥0.5 < 0.75		
	4	≥0.75 < 1		
	5	≥1		
Milk/Yoghurt	0	0	500	Upper limit represents the minimum number of serves recommended in the ADG.
	1	>0 ≤ 2 or >4		
	2	>2 ≤ 2.5		
	3	>2.5 ≤ 3		
	4	>3 ≤ 3.5		
	5	>3.5 ≤ 4		
Protein rich foods	0	0	335	Upper limit represents the minimum number of serves recommended in the ADG.
	1	>0 ≤ 2 or >3		
	2	>2 ≤ 2.25		
	3	>2.25 ≤ 2.5		
	4	>2.5 ≤ 2.75		
	5	>2.75 ≤ 3		
Starchy vegetables	0	0	335	Recommendation in HealthTrack study to limit to 1 serve per day.
	1	>0 ≤ 0.125 or >1		
	2	>0.125 ≤ 0.25		
	3	>0.25 ≤ 0.5		
	4	>0.5 ≤ 0.75		
	5	>0.75 ≤ 1		
Spreads/oils	0	0	200	Upper limit represents the maximum number of serves recommended in ADG. (allowance under Protein rich foods)
	1	>0 ≤ 2 or >4		
	2	>2 ≤ 2.5		
	3	>2.5 ≤ 3		
	4	>3 ≤ 3.5		
	5	>3.5 ≤ 4		
Alcoholic beverages	0	>2	10 g/d	Recommendation provided by the NHMRC.
	1	≤2		
Discretionary foods/beverages	0	>3	600	Allowance developed based on reported median intakes of “discretionary” items in HealthTrack study.
	1	>2 ≤ 3		
	2	>1 ≤ 2		
	3	≤1		

^a^Single serve equivalents determined using a published ready reckoner [59, 60]. Scoring criteria guided by ADG [8], NHMRC [58] and dietary advice provided in HealthTrack [48]

**Table 2:** Food groups categories used in the Diet Quality Tracker

Major food group classification in the DQT (*n* = 10)	Examples of sub-major food groups included
Grains	Refined grains
	Discretionary grains
	Non refined grains
	Mixed dishes where cereal is the major ingredient, e.g. pizza
Starchy vegetables	Starchy vegetables
	Mixed dishes where starchy vegetable is the major ingredient, e.g. potato salad
Legumes	Legumes
	Mixed dishes where legume is the major ingredient, e.g. falafel
Spreads/oils	Oils
	Spreads (including avocado)
Protein rich foods	Non oily fish
	Lean meat
	Low/reduced fat cheese
	Oily fish
	Soybean
	Medium fat meat
	Full fat cheese
	Egg
	Mixed dishes where protein rich food is the major ingredient, e.g. chicken casserole
Fruit	Fruit
	Dried fruit
	Juice
	Mixed dishes where fruit is the major ingredient, e.g. fruit crumble
Non-starchy vegetables	Non-starchy vegetables
	Mixed dishes where non-starchy vegetable is the major ingredient, e.g. pumpkin salad
Milk/Yoghurt	Low/reduced fat milk/yoghurt
	Full fat milk/yoghurt
	Milk alternatives e.g. soy milk
	Mixed dishes where milk/yoghurt is the major ingredient, e.g. smoothie
Discretionary foods/beverages	All foods/beverages categorised as “Discretionary” [57]
Alcoholic beverages	All alcoholic beverages

The DQT was applied to DH data at baseline, 3 and 12 months to obtain DQ scores which were analysed according to study group: intervention plus walnuts (*IW*), intervention (*I*) and control *(C).* Reported alcohol consumption was calculated as grams (g) of alcohol, in line with the National Health and Medical Research Council (NHMRC) definition [58]. Reporting of other foods and beverages were determined as the average daily contribution to energy intakes (kJ). A published ready reckoner [59, 60] developed using dietary trial data from the same region provided reference energy values to quantify the dietary data into single serving equivalents. A reference standard for daily reported consumption was applied to the food groups which reflected the range of minimum to maximum recommended number of servings listed in the ADG for healthy male and female adults under the age of 70 years [8]. The reference standards for food consumption were used for scoring diet quality (Table 1). Each ‘core’ food group was awarded 0–5 points if average daily intakes met respective standards for each food group. A dichotomous scoring criteria was used for alcohol consumption; one point was awarded if alcohol consumption did not exceed the NHMRC [58] recommendation of two standard drinks (20 g) and no points awarded if this criteria was exceeded. A reverse scoring system, and also a narrower range of 0–3 points, was used for ‘discretionary foods/beverages’ items. For the HealthTrack study DQT, higher scores reflected a higher diet quality (based on food quality) and inherently greater adherence to the ADG and the HealthTrack trial approach to dietary advice.

### A priori diet quality score

The APDQS [43–47, 61] consists of food groups postulated to be associated with reduced or increased risk of CVD. Food groups considered to be protective of CVD are classified as ‘positive’ (*n* = 25), while detrimental food groups are classified as ‘negative’ (*n* = 16) in the a priori diet score (Additional file 1: Table S1). A score of 0–4 was awarded to each of these food groups, with scores distributed across five levels of consumption criteria [61]. Scores were scaled in increasing order for ‘positive’ foods (i.e. greater consumption of ‘positive’ food groups was awarded greater points) and a reverse order for ‘negative’ foods (i.e lesser consumption of ‘negative’ food groups was awarded greater points). Final scores were calculated as the sum of ‘positive’ and ‘negative’ food groups. Food groups classified as ‘neutral’ (*n* = 13) were considered irrelevant to MI risk ([46] and thus did not contribute to the score.

To enable the a priori diet quality score to be applied to the HealthTrack study dietary data, serving sizes provided in the 2005 Dietary Guidelines for Americans [62] were used to determine servings per day. Conversion factors were applied to convert cups per day [62] to grams per day (g/d) [63], where required, as the former measure was not available for our data. Nut-based spreads, including peanut butter, were excluded from the “nuts” food group in the a priori as no serve recommendations were provided [62]. “Fatty fish” were classified in accordance with the method used by Neale et al [64].

### Statistical analysis

Dietary data utilised for these analyses was restricted to participants who completed the 12 month intervention period. De-identified data was analysed using IBM SPSS Statistics (Version 21, SPSS Inc, Chicago, Il, USA) with statistical significance was set at *P* < 0.05. Normality of data was assessed with Kolmogorov-Smirnov test and the distribution of data was further verified using histograms. Changes in diet quality score over time (baseline, 3 and 12 months) were assessed using two-way RMANOVA. A one-way ANOVA was also used to evaluate differences in the diet quality scores between the study groups at each time point, while a one-way RMANOVA tested for significant changes in scores over time for each group. Reported consumption of food groups, from both the DQT and APDQS, and nutrients were analysed using Kruskal-Wallis and Friedman’s tests. Post-hoc Bonferonni tests were conducted where statistically significant results were reported.

## Results

Complete data for *n* = 157 participants (*n* = 55 males; *n* = 102 females) was available for the analysis reported here. The DQ scores were noted to be parametric, however all other reported variables (demographics and dietary data) were largely non-parametric. Median (interquartile range [IQR]) age of study completers was 46 (38–51) years, weight 88.4 (79.2–101.4) kg and body mass index (BMI) 31.1 (28.6–34.0) kg/m^2^. DQ score outcomes (mean ± s.d) (Table 3) and dietary data (median and IQR) (Additional file 1: Table S2–S4) at baseline, 3 and 12 months have been presented. Post-hoc significant results have been denoted, and *p*-values reported below.

**Table 3:** Summary of diet quality score outcomes according to study group

	IW		I		C			*p*-value§		
	*n* = 67		*n* = 41		*n* = 49**					
Diet Quality Tracker scores	mean	s.d	mean	s.d	mean	s.d	*p*-value^†^	Time	Group	time*group
Baseline	19	4	19	4	19	5	0.902	**0.000**	0.233	**0.034**
3 months	22^a,y^	5	21^y^	4	19^a^	4	**0.007**			
12 months	20^z^	4	20	3	20	4	0.791			
*p-value* ^*‡*^	**0.000**		**0.002**		0.196					
a priori diet quality scores										
Baseline	83	12	83	14	82	14	0.861	**0.000**	**0.027**	**0.001**
3 months	96^b,y^	10	91^y^	13	87^b,y^	12	**0.001**			
12 months	91^c,y,z^	11	90^y^	12	84^c^	12	**0.006**			
*p-value* ^*‡*^	**0.000**		**0.000**		**0.023**					

**n = 47 at 3 months

^§^2-way RMANOVA

^†^1-way ANOVA

^‡^1-way RMANOVA

^a-c^ significant differences between groups

^y^ significant differences within groups from baseline

^z^ significant differences within groups from 3 months

*p*-values in bold indicate statistically significant values

### Changes in diet quality scores

#### Diet Quality Tracker

There was an overall effect on mean DQ scores over time, F(2304) = 2.632, *p* = 0.034, ƞ^2^ = 0.033 (Table 3), with higher mean *±* sd scores from *IW (22 ± 5)* compared to *C* (19 *±* 4) (p = 0.008) at 3 months. Scores changed significantly in the *IW* group, F(2132) = 11.492, *p* = 0.000, ƞ^2^ = 0.148, with higher scores achieved at 3 months compared to baseline (19 ± 4) (*p* = 0.000); however, scores decreased at 12 months (20 ± 4) compared to 3 months (*p* = 0.033). The *I* group also reported a significant change in scores, F(2,80) = 6.708, *p* = 0.002, ƞ^2^ = 0.144, with an improvement at 3 months (21 *±* 4) from baseline (19 *±* 4) (*p* = 0.006). No significant change in diet quality scores were reported for the *C* group.

#### A priori diet quality score

Similar to the DQT, an overall effect on mean diet quality scores over time, F(2304) = 4.406, *p* = 0.001, ƞ^2^ = 0.058 (Table 3) was reported from the a priori diet quality score. At 3 months, the *IW* group recorded higher scores (96 ± 10) compared to *C* (87 ± 12) (*p* = 0.000), with similar findings at 12 months (*IW*: 91 ± 11, *C*: 84 ± 12; *p* = 0.006). Scores also changed significantly for the *IW* group, F(2132) = 53.220, *p* = 0.000, ƞ^2^ = 0.446, with higher scores at 3 and 12 months compared to baseline (*p* = 0.000 for both), but a lower score at 12 months compared to 3 months (*p* = 0.001). Scores for the *I* group, F(2,80) = 12.238, *p* = 0.000, ƞ^2=^0.234, increased at 3 (91 ± 13, *p* = 0.000) and 12 months (90 ± 12, *p* = 0.002) compared to baseline (83 ± 14). The *C* group also reported a change in scores, F(2,92) = 3.942, *p* = 0.023, ƞ^2=^0.079, with higher scores achieved at 3 months (87 ± 12) compared to baseline (82 ± 14) (*p* = 0.044).

### Changes in food choices

#### Diet Quality Tracker (Additional file 1: Table S2)

Discretionary foods/beverage reporting reduced in all groups after 3 (*IW* and *I, p* = 0.000, *C p* = 0.003) and 12 months (*IW* and *I p* = 0.000, *C p* = 0.012), with *IW* consuming less than *C* (*p* = 0.027) at 3 months. Reported consumption of nuts/seeds increased in the *IW* group at 3 (*p* = 0.000) and 12 months (*p* = 0.001) compared to baseline, although 12 month consumption was lower than 3 months (*p* = 0.047). Nuts/seeds consumption for *IW* was also greater than *I* and *C* groups at 3 (*P* = 0.000) and 12 months (*P* = 0.003). The *IW* (*P* = 0.002) and *C* (*P* = 0.000) groups decreased reported grain consumption at 3 months, a trend which continued for *C* at 12 months (*P* = 0.008). The *I* group reported reduced consumption of non-starchy vegetables at 12 months (*P* = 0.033), while *IW* reported consuming more non-starchy vegetables than *C* (*P* = 0.029) at 3 months. On the other hand, *C* reported more protein-rich food than the *IW* group (*P* = 0.009) who consumed lower amounts at 3 (*P* = 0.001) and 12 months (*P* = 0.029) compared to baseline intakes. Although the *I* group also reported less protein-rich foods (*P* = 0.012), in addition to lower alcohol consumption (*P* = 0.017) at 3 months, however, intakes of fruit (*P* = 0.017) at 3 months as well as spreads and oils at 12 months (*P* = 0.024) were higher than at baseline. Consumption of fruit and starchy vegetables were also reported to be significantly different for *IW* over time, but significance was no longer detected after conducting post-hoc tests.

#### A priori food diet quality score (Additional file 1: Table S3)

Similar to the changes seen in the DQT nuts/seeds food group, intake of nuts (g/d) increased for *IW* at 3 and 12 months (*P* = 0.000 for both). Although nut consumption in the *IW* group decreased from 3 to 12 months (*P* = 0.009), the *IW* group reported the highest nut consumption of all groups at 3 (*P* = 0.000) and 12 months (*I: P* = 0.000, *C*: *P* = 0.002). There were also similar trends across all groups with changes over time in consumption of some food groups. All groups decreased grain based desserts at 3 and 12 months (*P* = 0.000 for all). All groups also reported less refined grains consumption (*IW*: *P* = 0.000, *I*: *P* = 0.004, *C*: *P* = 0.001) at 3 and 12 months (*IW*: *P* = 0.002, *I*: *P* = 0.045, *C*: *P* = 0.016), with *IW* consuming lower amounts than *C* (*P* = 0.021) at 3 months. Consumption of salty snacks decreased for *IW* (*P* = 0.000) at 3 months, with *IW* consuming less salty snacks than *I* at 3 months (*P* = 0.046). However, at 12 months, salty snack consumption decreased for all groups (*IW*: *P* = 0.000, *I*: *P* = 0.001, *C*: *P* = 0.030), and for *I*, this was also a significant decrease compared to reported intakes at 3 months (*P* = 0.020).

There were changes in consumption of other food groups at 3 months. Both *IW* and *I* increased consumption of other vegetables (*IW*: *P* = 0.000, *I*: *P* = 0.045) and fruit (*IW*: *P* = 0.000, *I*: *P* = 0.003). The *IW* group increased consumption of tomato (*P* = 0.005) and low fat milk (*P* = 0.025), while consumption of poultry (*P* = 0.037), fried potato (*P* = 0.003) and chocolate (*P* = 0.001) decreased. The *IW* group also consumed more low fat yoghurt (*P* = 0.000) compared to baseline, and consumed higher amounts of low fat yoghurt than the *I* (*P* = 0.003) and *C* (*P* = 0.001) groups at 3 months. The *I* group consumed less lean meats (*P* = 0.004) but more wholegrain bread (*P* = 0.003) compared to baseline. The *IW* (*P* = 0.032) and *I* (*P* = 0.036) groups consumed more wholegrain cereal than *C,* who also ate more confectionery (*P* = 0.026) than at baseline.

At 12 months the *IW* group increased consumption of confectionery (*P* = 0.005) and continued to consume more low fat yoghurt (*P* = 0.041), but decreased intakes of tomato (*P* = 0.047), fried potato (*P* = 0.047), lean meats (*P* = 0.041) and chocolate (*P* = 0.000). The *C* group decreased coffee consumption (*P* = 0.030) compared to baseline intakes. The *IW* and *I* groups also consumed reduced amounts of other vegetables (*P* = 0.001), with the *I* group eating less tomato in comparison to intakes at 3 months (*P* = 0.045), but more soup than *IW* (*P* = 0.039).

There were differences between the groups in consumption of eggs and sugar substitutes at baseline, fruit, soy products and pickled foods at 3 months, full fat milk and soft drinks at 12 months, but no post hoc significance was detected. Furthermore, although there were reported differences in intakes of avocado, soft drinks, eggs and sugar substitutes for *IW*, fried potato, full fat milk and cheese for *I*, and other vegetables for *C,* these were not significant from post hoc tests. Lastly, although there were reported differences in pastry consumption by both *IW* and *I*, no post hoc significance was detected for reported consumption of these food groups.

### *Changes in nutrient parameters* (Additional file 1: Table S4)

Reported energy intake decreased for *IW* and *I* at 3 and 12 months (*P* = 0.000). Reported dietary fibre decreased for *IW* at 12 months compared to 3 months (*P* = 0.029). The percentage of energy from carbohydrates decreased at 12 compared to 3 months (*P* = 0.024) for *I.* In comparison, while reductions in percentage energy from carbohydrate were also found for *IW*, these results were no longer significant after applying post-hoc tests.

There were notable changes to dietary fat profile. The *IW* group reported the highest percentage energy from fat at 3 months compared to *I* (*P* = 0.002) and *C* (*P* = 0.031), while this decreased for *I* from baseline to 3 months, but increased from 3 to 12 months (*P* = 0.001 for both). A decrease in the proportion of fat as monounsaturated fatty acid (MUFA) was reported from baseline at 3 (*P* = 0.000) and 12 months (*P* = 0.006) for *IW*, but this rebounded at 12 months compared to 3 months (*P* = 0.006). In comparison to *I* and *C*, *IW* reported lower dietary fat proportions as MUFA at 3 (*P* = 0.000) and 12 months (*I*: *P* = 0.001, *C*: *P* = 0.000). The proportion of fat as polyunsaturated fatty acids (PUFA) and the polyunsaturated fatty acid: saturated fatty acid (P:S) ratio was higher for *IW* than *I* and *C* at 3 and 12 months (*P* = 0.000). Intakes of PUFA increased at 3 months for *IW* (*P* = 0.000) and *C* (*P* = 0.022), which remained high at 12 months for *IW* (*P* = 0.000). However, although PUFA intakes for *IW* remained highest compared to *I* and *C* at 12 months (*P* = 0.000), this was lower than at 3 months (*P* = 0.000). In all three study groups, the proportion of reported fat as saturated fatty acids (SFA) decreased at 3 months (*IW: P* = 0.000, *I: P* = 0.012 and *C: P* = 0.022). Consumption of fat as SFA continued to decrease for *IW* (*P* = 0.000) and *I* (*P* = 0.017) at 12 months although this was higher than at 3 months for *IW* (*P* = 0.000). However, despite some rebound, *IW* reported the lowest consumption of fat as SFA at 3 (*P* = 0.000) and 12 months (*I: P* = 0.027, *C*: *P* = 0.000) among the groups.

## Discussion

In the intensive phase of the trial (0,1,2 3 months), intervention participants who received individualised counselling sessions with dietitians produced greater improvements in diet quality. Walnut supplementation enhanced this effect and we were able to demonstrate this using a brief index (DQT), as well as a validated tool (APDQS). Both indices provided evidence that improvements were greatest at 3 months. Even though all groups reduced consumption of foods deemed ‘discretionary’ (poor nutritional quality), the APDQS which had greater specificity at the food group level was able to reveal which food groups in particular were affected. The increased consumption of nuts in the *IW* group was reflected in changes to dietary fat, namely decreased SFA and MUFA, while PUFA and P:S ratio increased. As the trial progressed to less frequent clinic visits, there was some variation in these patterns, as would be expected, but overall the impact of individualised advice and food supplementation meant the *IW* group continued to fare best.

### Changes in food choices

Changes in food choices were consistent with results from other studies in which higher diet quality scores reflected lower consumption of meat [5, 65, 66], alcohol [33, 67] and discretionary food items [29, 31]. The observed increased consumption of fruit (found with APDQS and DQT), non-starchy vegetables (found with DQT), tomato and other vegetables (found with APDQS) was also congruent with other studies associating higher intakes of vegetables and fruits associated with high diet quality [65, 66]. Importantly reductions in foods considered ‘discretionary’ in the DQT were ‘negative’ foods listed in the APDQS, notably, salty snacks and grain based desserts. These findings reflected the different degrees of specificity of the indices applied, however the only real ambiguity was how best to represent alcohol consumption. The DQT utilised a reverse scoring system for alcoholic beverages as this was discouraged in the HealthTrack study; but, the a priori diet quality score allocates increasing points with respect to higher intakes. In our analysis, however, only the *I* group decreased alcoholic beverages at 3 months, so the absence of significant changes in beer, wine and liquor/spirits intakes as distinct food groups in the APDQS may have had little impact on the total a priori diet quality score.

Evaluating change in consumption of grains is also problematic given the need to include moderate amounts and there are differences in quality of grains. In the trial context, we found decreased intakes of grain foods as have others [5], but not all [33, 66–68]. In these latter studies, reporting high intakes of grain based foods from wholegrain sources was representative of higher diet quality. In our study, lower intakes of grain based foods did not reduce diet quality but this may reflect synergies with other food groups assessed in the DQT. Alternatively, as the DQT did not distinguish between wholegrain and non-wholegrains foods (in keeping with the AGHE), the tool may be limited in valuing grain intake. Nevertheless, as the DQT was guided by a combination of dietary considerations, improvements to diet quality scores would be indicative of better adherence towards to the AGHE, and hence, a healthier pattern of food consumption. On the other hand, wholegrain cereal, bread and rice/pasta were scored as ‘positive’ food groups in the a priori diet quality score. Using the APDQS we were able to distinguish that significantly more wholegrain cereal was consumed by both intervention arms compared to the *C* group at 3 months, while the *I* group consumed significantly more wholegrain bread. Although refined grains are considered a ‘neutral’ food group and did not contribute to the final a priori diet quality score, it was also noted all groups ate less refined grains in contrast to baseline intakes, a further reflection of improvements to overall diet quality.

Regardless of the index used the high diet quality scores for *IW* is consistent with studies when walnuts and nuts more generally were provided in controlled portions [69–74]. It has been suggested that providing nuts may improve diet quality by displacing consumption of other less nutritious foods [70]. Nuts are characteristically satiating [75, 76] and although a high energy dense food, 55–75% of energy contributed from nuts appears to be offset through compensatory metabolic responses [77]. The consumption of walnuts (and other nuts) is associated with cardio-protective effects [78, 79] including improving serum lipid profiles [80] without promoting weight gain [81]. Providing these foods in trial settings may support adherence towards dietary interventions [69–74] provided quantities for consumption were deemed acceptable [72]. At least in the trial setting, providing a healthy food supplement, such as walnuts, in trials appears to lead to superior adherence and improvements in diet quality outcomes. In this study all groups were encouraged to reduce discretionary foods and it appears this advice was followed. A significant finding, however, was that the addition of walnuts in the *IW* group appeared to aid in increased consumption of vegetables, low fat milk and yoghurt, and less protein rich foods than the *I* and *C* groups, which may in itself have advantages [82–85].

### Changes in nutrient parameters

Changes to the nutrient profiles in the *IW* group also reflected findings in other nut studies, notably significant increases to the percentage of total reported energy as fat [71, 86] and fat as PUFA [70–72, 86]. Decreases in percentage of fat as SFA [71, 86] and MUFA [87] may have resulted from increased walnut consumption by the *IW* group but also correspond with lower intakes of protein rich foods in this study, as MUFAs tend to be found with food sources containing SFA, i.e. animal-based meats and plant oils [88]. The decrease in dietary MUFA was consistent with another study which also included 30 g/d of walnuts [87], but other studies of other nuts reported increases in fat as MUFA [71, 72, 89], reflecting variations in food composition by nut variety: different nuts were provided in these instances, hazelnuts [71, 72] and almonds [89]. Hazelnuts are four times richer in MUFA, but contain seven times less PUFA than walnuts per 100 g [90] while almonds have three time more MUFA but three times less PUFA per 100 g compared to walnuts [90]. Another significant nutrient change for the *IW* group was an increase in P:S ratio, corresponding to increases in PUFA and decreases in SFA, and this could be attributed to the substitution of other foods for nuts/seeds compared to the other two study groups.

In studies where nut consumption was associated with higher energy intakes [70, 72, 86, 89], they were not designed as weight loss interventions. Lower energy intakes in HealthTrack may have resulted from adherence to reduced energy diet plans provided to all participants, similar to other behavioural weight loss intervention programs with the inclusion [87] or exclusion of nuts [5, 6]. Improvements in the diet quality of control groups participating in behavioural interventions is not an unusual occurrence [91, 92], a phenomenon which could result from increased awareness by participating in a trial [92]. Thus, in our study, it could be surmised that reductions in reported energy intake was a result of changes in diet related behaviours, including less discretionary foods/beverage consumption. This is an encouraging observation, particularly as over-consumption of discretionary foods and/or beverages is recognised as an indicator of poor diet quality [93, 94], and leads to weight gain [94–97].

### Limitations

Even though we applied a validated diet quality index, our analysis was based in part on the development of a context specific DQT, and designing these tools requires subjective decisions [16]. One of our decisions was to not score the nuts/seeds food group because one of the treatment arms was given supplements which could have created a bias in favour of the *IW* group. In addition, a range of points were allocated for food groups indicative of dietary guidelines, but a narrower range of points were applied to discretionary food items to emphasise dietary guidance to limit the frequency of consumption of these foods. The median intakes of discretionary foods by the study sample were used to develop the cut-off values.

In using the two diet quality tools we were also aware that there were differences in the units of measurement between the DQT (kJ/d) and the a priori diet quality score (g/d). Additionally, in the DQT, alcoholic beverage consumption was measured as g/d of alcohol for consistency with the NHRMC [58] guidelines, while the US dietary guidelines [62] references volume of the beverage as a serve guide. Therefore, while direct comparisons of median values of reported consumption between the DQT and a priori diet quality score may not be appropriate, changes in trends may still be evaluated.

Strategies adopted when promoting healthy eating focus on increasing or decreasing certain foods groups, and within clinical weight loss settings, dietary recommendations typically emphasise the inclusion of a range of nutritionally sound foods, and an ideal healthy pattern of foods [9]. The dietary advice provided to intervention groups in the HealthTrack study reflected advice provided by dietitians in a clinical setting. The focus on foods, and not nutrients, supported the holistic principles of dietary pattern analyses [6, 20, 22, 98]. Failure to achieve levels recommended in dietary guidelines, however, suggests it may be more difficult to make the necessary changes to certain food groups than others [91]. Although median intakes of foods considered ‘discretionary’ decreased significantly for HealthTrack participants, it remained above the AGHE recommended single serve providing 600 kJ. In addition, legume consumption was low across the sample despite advice provided to the intervention arms to include this food group. This study demonstrated that overall DQ is represented by the additive effect of a combination of several food groups (i.e. food synergy). It affirms the value of considering individual food consumption together with nutrient intakes, while recognising the significance of certain food choice patterns for implementing beneficial dietary changes. The translation to practice is tangible as humans eat foods, not isolated nutrients or single foods [16].

A final limitation relates to the general problem of dietary data. The data used in the analyses were self-reported, and misreporting errors are inherent to DH [99, 100]. DH are reliant on recall abilities of study participants [100, 101], thus misreporting errors may arise from recall failure, inaccuracies, or from under or over-reporting usual intakes [102]. Nevertheless, where diet quality research is concerned, self-reported dietary data continues to provide the required detail to support analysis for overall patterns of consumption [103].

## Conclusions

Lifestyle intervention targeting weight loss that focused on Dietary Guidelines also improved diet quality, as determined with a validated DQI, and a tool specifically designed for the HealthTrack study. Whilst minor differences in food group intakes were detected between tools (which could be attributed to methodological differences), the overall patterns of changes in diet quality were similar. The improvements in diet quality could be attributed to the message to limit ‘discretionary’ foods which then had an impact on food choice patterns and helped to reduce energy intakes. The impact between general and individualised Dietary Guidance advice was better exposed when the latter was supplemented with walnuts, an example of a healthy food. This also resulted in improved dietary fat profile, and a greater consumption of nuts and vegetables, with less meat in the overall dietary pattern. Thus the introduction of a healthy food supplement (walnuts) resulted in relatively greater improvements in overall diet quality, seen through broader shifts in dietary habits. In summary, this analysis showed that reducing energy intakes can be accompanied by increased diet quality, but the approach to dietary guidance is an important consideration.

## Additional file

Additional file 1: **Table S1.** List of food groups classified as 'positive', 'negative' and 'neutral' in the *a priori diet quality score* (APDQS). This file contains the list of food groups classified as ‘positive, ‘negative’ and ‘neutral, in the a *priori diet quality score* (APDQS). **Table S2.** Median daily consumption of food groups from the Diet Quality Tracker (DQT). This file contains data relating to the median daily consumption of food groups used in the Diet Quality Tracker (DQT). **Table S3.** Median daily consumption of food groups from the *a priori diet quality score* (APDQS). This file contains data relating to the median daily consumption of food groups used in the *a priori diet quality score* (APDQS). **Table S4.** Median daily nutrient intakes as reported in diet histories. This file contains data relating to the median daily nutrient intakes from the HealthTrack study diet history data used for analysis of this study. (XLSX 50 kb)

## Abbreviations

ADG: Australian Dietary Guidelines
AGHE: Australian Guide to Healthy Eating
AHS: Australian Health Survey
APDs: Accredited Practising Dietitians
AUSNUT: Australian Food and Nutrient
BMI: Body mass index
C: Control group
CVD: Cardiovascular disease
DH: Diet history
DQ: Diet quality
DQIs: Diet quality indices
DQT: Diet Quality Tracker
I: Intervention only group
IQR: Interquartile range
IW: Intervention plus walnuts group
MUFA: Monounsaturated fatty acid
NHMRC: National Health and Medical Research Council
NNPAS: National Nutrition and Physical Activity Survey
P:S: Polyunsaturated fatty acid: saturated fatty acid
PUFA: Polyunsaturated fatty acids
RCT: Randomised controlled trial
SFA: Saturated fatty acids.
